# A sheep in wolf's clothing: *Elaphe xiphodonta* sp. nov. (Squamata, Colubridae) and its possible mimicry to *Protobothrops jerdonii*

**DOI:** 10.3897/zookeys.1048.65650

**Published:** 2021-07-06

**Authors:** Shuo Qi, Jing-Song Shi, Yan-Bo Ma, Yi-Fei Gao, Shu-Hai Bu, L. Lee Grismer, Pi-Peng Li, Ying-Yong Wang

**Affiliations:** 1 State Key Laboratory of Biocontrol/ The Museum of Biology, School of Life Sciences, Sun Yat-sen University, Guangzhou, Guangdong 510275, China Sun Yat-sen University Guangzhou China; 2 Institute of Herpetology, Shenyang Normal University, Shenyang 110034, China Shenyang Normal University Shenyang China; 3 Key Laboratory of Vertebrate Evolution and Human Origins of Chinese Academy of Sciences, Institute of Vertebrate Paleontology and Paleoanthropology, Chinese Academy of Sciences, Beijing 100044, China Institute of Vertebrate Paleontology and Paleoanthropology, Chinese Academy of Sciences Beijing China; 4 College of Life Sciences, Northwest Agriculture and Forestry University, Yangling, 712100, China Northwest Agriculture and Forestry University Yangling China; 5 Herpetology Laboratory, Department of Biology, La Sierra University, Riverside, California 92515, USA La Sierra Univer­sity Riverside United States of America

**Keywords:** Colubrid, morphology, osteology, Qinling Mountains, taxonomy

## Abstract

Based on combined morphological and osteological characters and molecular phylogenetics, we describe a new species of the genus *Elaphe* that was discovered from the south slope of the Qinling Mountains, Shaanxi, China, namely *Elaphe xiphodonta***sp. nov.** It is distinguished from the other congeners by a combination of the following characters: dorsal scales in 21-21-17 rows, the medial 11 rows keeled; 202–204 ventral scales, 67–68 subcaudals; two preoculars (including one subpreocular); two postoculars; two anterior temporals, three posterior temporals; reduced numbers of maxillary teeth (9+2) and dentary teeth (12); sharp cutting edges on the posterior or posterolateral surface of the rear maxillary teeth and dentary teeth; dorsal head yellow, three distinct markings on the head and neck; a distinct black labial spot present in supralabials; dorsum yellow, 46–49 complete (or incomplete) large black-edged reddish brown blotches on the body and 12–19 on the tail, two rows of smaller blotches on each ventrolateral side; ventral scales yellow with mottled irregular black blotches, a few irregular small red spots dispersed on the middle of the ventral. Based on molecular phylogenetic analyses, the new species forms the sister taxon to *E. zoigeensis*. The discovery of this new species increases the number of the recognized species in the genus *Elaphe* to 17.

## Introduction

The colubrid genus *Elaphe* sensu lato Fitzinger (in Wagler), 1833, once contained approximately forty species ranging throughout temperate, subtropical, and tropical zones in both the eastern and western hemispheres (Schulz 1996). Most of the members of this genus have a slender body, partially or completely keeled dorsal scales, and round pupils. The head is distinguishable from the neck, the trunk vertebra lack a hypapophysis, and the posterior maxillary teeth are not significantly differentiated from the others. With the rise of molecular taxonomy in the last decades of the 20^th^ century, a series of major taxonomic changes occurred at generic and species levels, resulting in the establishment or resurrection of several genera and species (Helfenberger 2001; Lenk Joger and Wink 2001; Utiger et al. 2002; Huang et al. 2012; Jablonski et al. 2019). Recent molecular phylogenetic studies suggest that the genus *Orthriophis* should be subsumed within the genus *Elaphe*, because the generic status of *Orthriophis* renders *Elaphe* paraphyletic, where *E. zoigeensis* Huang, Ding, Burbrink, Yang, Huang, Ling, Chen & Zhang, 2012 is sister to all the other *Elaphe* plus *Orthriophis* (Chen et al. 2017; Li et al. 2020; but see Figueroa et al. 2016). Currently, the genus *Elaphe* sensu stricto is comprised of 16 species of which most, are widely distributed in eastern Asia and the south slopes of the Himalaya, although the range of the genus extends east to eastern Russia, south to the Indonesia-Malayan region, and west to as far as Italy (Helfenberger 2001; Zhao 2006; Huang et al. 2012; Jablonski et al. 2019; Uetz et al. 2021). Previous biogeographic, phylogenetic, and phylogenomic studies support the hypothesis that *Elaphe* originated in the Eastern Palearctic (Lenk, Joger and Wink 2001; Utiger et al. 2002; Burbrink and Lawson 2007; Burbrink and Pyron 2010; Chen et al. 2017). In regards to China, 11 species of *Elaphe* are recognized: *E. anomala* (Boulenger, 1916), *E. bimaculata* Schmidt, 1925, *E. carinata* (Günther, 1864), *E. cantoris* (Boulenger, 1894), *E. davidi* (Sauvage, 1884), *E. dione* (Pallas, 1773), *E. hodgsonii* (Günther, 1860), *E. moellendorffi* (Boettger, 1886), *E. taeniura* (Cope, 1861), *E. schrenckii* (Strauch, 1873), and *E. zoigeensis*, two of which (*E. bimaculata* and *E. zoigeensis*) are endemic to China (Wang et al. 2020).

The main part of Qinling Mountains, lies on the south of Shaanxi Province, having an average elevation of approximately 2000 m and have long been regarded as the geographical, biological and climatological boundary between North (Palaearctic Realm; warm temperate monsoon climate) and South China (Oriental Realm; subtropical monsoon climate, Zhang 1999). Due to the unique environment and climate, the Qinling Mountains are the habitat of many rare animals (e.g., *Ailuropoda melanoleuca*, *Budorcas bedfordi*, and *Rhinopithecus roxellana qinlingensis*). Additionally, the herpetological diversity of that area is high. To date, more than 10 species of amphibians and 20 species of reptiles have been reported in this area, some of which are endemic to the Qinling Mountains and adjacent areas (e.g., *Scutiger ningshanensis*, *Hyla tsinlingensis*, *Batrachuperus taibaiensis*, *Stichophanes ningshaanensis*, *Scincella tsinlingensis*, *Protobothrops jerdonii* and *Gloydius qinlingensis* (Bu and Zheng 2015).

Bates (1862) discovered a spectacular type of adaptation known as “mimicry”, building on Charles Darwin's views on evolution. This phenomenon, now called “Batesian mimicry”, which is defined as an edible species (mimic) evolving to resemble a conspicuous inedible species (model), thereby gaining protection from predation, its efficiency relying on confusing the mimic with the model (Carpenter and Ford 1933; Ruxton et al. 2004). Batesian mimicry is observed among a wide variety of animals, ranging from invertebrates to vertebrates, including several non-venomous snakes mimicking the color pattern, head shape or behavior of sympatric venomous snakes to avoid predation (Brodie 1993).

**Figure 1.**
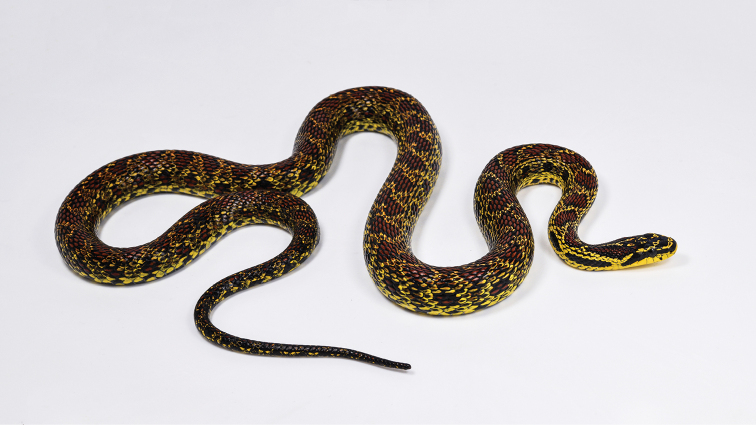
General view of the holotype (SYS r002534) of *Elaphe xiphodonta* sp. nov. in life. Photo by Shuo Qi.

**Figure 2.**
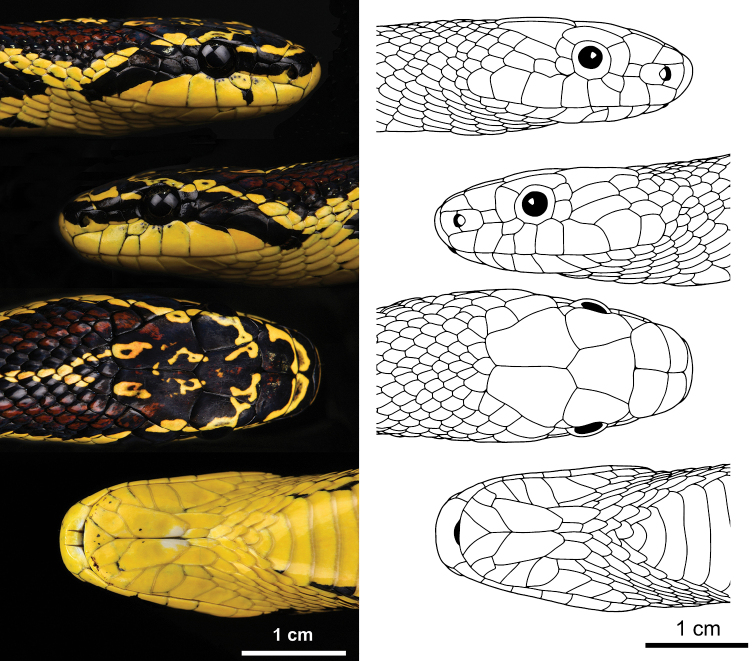
Detailed pholidosis of the head of the holotype (SYS r002534) of *Elaphe xiphodonta* sp. nov. Photos by Shuo Qi, illustrated by Xue-Man Zheng.

**Figure 3.**
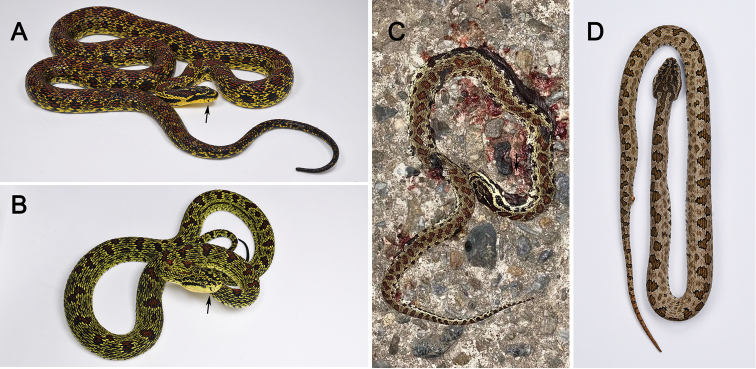
Comparison between *Elaphe xiphodonta* sp. nov. and sympatric *Protobothrops jerdonii* in different age stages **A** adult *Elaphe xiphodonta* sp. nov. (SYS r002534, holotype) **B** adult *Protobothrops jerdonii***C** juvenile *E. xiphodonta* sp. nov. (IVPP OV 2721, paratype), road-killed specimen **D** juvenile *P. jerdonii* specimen in preservative. The black arrow points to the labial spot. Photos **A, B, D** by Shuo Qi, photo **C** by Liang Sun.

During our recent herpetological surveys in the south slope of the Qinling Mountains, Shaanxi, China, two colubrid specimens were collected, which look quite different to any of the known species but similar to *Protobothrops jerdonii* (Figs 1–3). Detailed morphological examinations and further molecular analyses revealed that these specimens represent a separately evolving lineage within the genus *Elaphe* and can be distinguished from all congeners by morphological characters. We herein describe this overlooked *Elaphe* population as a new species. Furthermore, we suspect this new species is able to avoid predation by mimicking the syntopic pit-viper (*P. jerdonii*).

## Materials and methods

### Morphometrics

**Figure 4.**
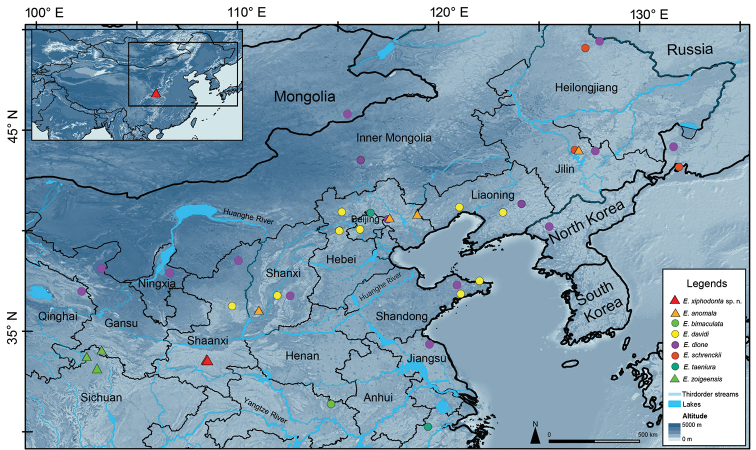
Type terra of *Elaphe xiphodonta* sp. nov (marked with red triangles), with the collection localities of some other Chinese species of *Elaphe*.

Morphological examinations were performed on two specimens collected from Chengguan Town, Ningshaan County, Shaanxi Province, China (Fig. 4). Both specimens were fixed in 10% buffered formalin after taking the tissue samples (liver or muscle), and then transferred to 70% ethanol for permanent preservation. The specimens are deposited in the Museum of Biology, Sun Yat-sen University (SYS r002534, Figs 1, 2, 3A) and Institute of Vertebrate Paleontology and Paleoanthropology, Chinese Academy of Sciences (IVPP OV 2721, Fig. 3C).

Morphological descriptions follow Dowling (1951) and Zhao (2006). The following measurements were taken with digital calipers (Neiko 01407A Stainless Steel 6-Inch Digital Caliper, USA) to the nearest 0.1 mm: **TL** total length (from tip of snout to tip of tail); **SVL** snout-vent length (from tip of snout to posterior margin of cloacal plate); **TaL** tail length (from posterior margin of cloacal plate to tip of tail); **HL** head length (from tip of snout to posterior margin of the mandible); **HW** maximum head width; **ED** eye horizontal diameter; **RW** = maximum rostral width; **RH** maximum rostral height.

Scalation features and their abbreviations are as follows: **DSR** dorsal scale rows, counted at one head length behind head, at midbody, and at one head length before vent; **SPL** supralabials; **IFL** infralabials; **CS** chin shields; **PrO** preoculars; **PtO** postoculars; **LoR** loreal; **aTMP** anterior temporals; **pTMP** posterior temporals; **PrV** preventral scales; **V** ventral scales; **PrC** precloacal plate; **SC** and subcaudals. Gender was determined by dissection or by the presence/absence of everted hemipenes. The numbers of maxillary teeth (**MT**) were counted based on the three-dimensional reconstructed model.

Other morphological characters (e.g., coloration, scalation, and size) for *Elaphe* taxonomy were obtained from Boulenger (1894), Stejneger (1907), Wen and Ji (1997), Zhao (2006), Huang et al. (2012), Jablonski et al. (2019), Shi et al. (2019) and Che et al. (2020).

The following abbreviations for museum collections are used throughout the paper:

**AMNH**American Museum of Natural History;

**BFU** Beijing Forest University;

**BM** British Museum;

**CAS** Chinese Academic of Sciences;

**IVPP** Institute of Vertebrate Paleontology and Paleoanthropology;

**MHNG**Muséum d’Histoire Naturelle, Genève;

**SYS** Sun Yat-sen University.

### X-ray scanning and three-dimensional reconstructions

The X-ray scanning was carried out with Nano-computerized tomography. Specimens were scanned using a GE v|tome|x m dual tube 300/180kV system in the Key Laboratory of Vertebrate Evolution and Human Origins, Institute of Vertebrate Paleontology and Paleoanthropology (IVPP), Chinese Academy of Sciences. The specimen was scanned with an energy beam of 80 kV and a flux of 80*μA using a 360° rotation and then reconstructed into the 4096*4096 matrix of 1536 slices. The final CT reconstructed skull images were exported with a minimum resolution of 6.099 μm. The skull images were exported from the virtual 3D model which was reconstruct by Volume Graphics Studio 3.0.

The dataset of the 3D model included in this study is available online in the repository (ADMorph, Hou et al. 2020) at http://www.admorph.org/; https://doi.org/10.12112/R.0005 (IVPP OV 2721, paratype).

### DNA Extraction, Polymerase Chain Reaction (PCR) and sequencing

For the molecular analyses, two tissue samples of the new species were included, which were taken prior to fixation, preserved in 99% alcohol, and stored at -40 °C.

Genomic DNA was extracted from muscle or liver tissue samples, using a DNA extraction kit from Tiangen Bio-tech (Beijing) Co., Ltd. Partial segments of the mitochondrial genes, 16S ribosomal RNA gene (16S), cytochrome C oxidase 1 gene (CO1) and Cytochrome b gene (cytb) were amplified. Nested PCR experiments were performed as described in Li et al. (2017). Primers used for PCR and sequencing followed Li et al. (2020), see Table 1 for details. The first PCR procedure was performed with an initial denaturation at 94 °C for 4 min, 35 cycles of 94 °C for 45 s, 45 °C for 40 s and 72 °C for 2 min, followed by a final extension at 72 °C for 10 min. The second PCR procedure was performed with an initial denaturation at 94 °C for 4 min, 30 cycles of 94 °C for 45 s, 50 °C for 40 s and 72 °C for 1.5 min, followed by a final extension at 72 °C for 10 min. PCR products were purified with spin columns and then sequenced using BigDye Terminator Cycle Sequencing Kit as per the guidelines on an ABI Prism 3730 automated DNA sequencer by Guangzhou Tianyi Huiyuan Bio-tech Co., Ltd.

**Table 1.** Nested PCR primers for this study (Li et al. 2020).

Gene	Primer name	Assay	Sequence
16S	12S-16S_Phe_F1	1^st^PCR	AAGCDYDGCRCTGAAAATGC
	12S-16S_ND1_R1		AANGCNACNGCDATNAR
	S12S902L2	2^nd^PCR	YACACACCGCCCGTCA
	S16S2984H2		GACCTGGATTDCTCCGGTCTGAACTC
COI	COI_Asn_F1	1^st^PCR	GDTTRGRYKDYTARYTGTTAAYTA
	COI_Asp_R1		GTDATTYRRYYDYGACA
	COI_25_F2	2^nd^PCR	TCRACHAAYCAYAAAGAYATYGG
	COI_706_R2		TADACTTCWGGRTGDCCRAARAATCA
cytb	Scytb15025F	1^st^PCR	TGGTGGAAYTTYGGNWSNATGYT
	Scytb15726R		TANGCRAANARRAARTACCAYTC
	Scytb15082F	2^nd^PCR	TTYTTYYTRGCNRTHCAYTAYAC
	Scytb15692R		GCYTTDVWRAARTTKTCNGGRTC

### Phylogenetic analyses

Fifty-nine sequences from 16 known *Elaphe* species plus six outgroup sequences from *Euprepiophis mandarinus* (Cantor, 1842) used to root the tree, were obtained from GenBank, and composed the dataset (Table 2).

**Table 2.** Localities, specimen vouchers and GenBank accession numbers of the specimens included in this study.

No.	Species name	Locality	Specimen voucher	Genbank accession number			References
				16S rRNA	CO1	Cytb	
**1**	*Elaphe xiphodonta* sp. nov.	Ningshaan, Shaanxi, China	SYS r002534	MZ24210000	MZ19164	MZ19166	This study
**2**		Ningshaan, Shaanxi, China	IVPP OV 2721	MZ242101	MZ19165	MZ19167	This study
**3**	*Elaphe anomala*	Huangshan, Anhui, China	HS11075	MK193929	MK064632	MK201281	Li et al. 2020
**4**	*Elaphe bimaculata*	Huangshan, Anhui, China	HS15168	MK193931	MK064634	MK201283	Li et al. 2020
**5**	*Elaphe cantoris*	Pailong, Tibet, China	JK201705	MK194263	MK064913	MK201564	Li et al. 2020
**6**	*Elaphe carinata*	Guangze, Fujian, China	HS13055	MK193932	MK064635	MK201284	Li et al. 2020
**7**		Longyou, Zhejiang, China	HS13062	MK193934	MK064637	MK201286	Li et al. 2020
**8**	*Elaphe climacophora*	Abashiri, Hokkaido, Japan	KUZ R64481	N/A	LC328423	LC327534	Moriyama et al. 2018
**9**		Deshikutsu, Hokkaido, Japan	KUZ R68813	N/A	LC328426	LC327537	Moriyama et al. 2018
**10**	*Elaphe davidi*	Taishan Mt., Shandong, China	N/A	KM401547	KM401547	KM401547	Xu et al. 2015
**11**	*Elaphe dione*	Taibai, Shaanxi, China	HS11036	MK193928	MK064631	MK201280	Li et al. 2020
**12**	*Elaphe hodgsonii*	Jilong, Tibet, China	HS13004	MK193983	MK064680	MK201335	Li et al. 2020
**13**	*Elaphe moellendorffi*	Yunlin, Guangxi, China	S-113	N/A	KF698944	KF913314	Cao et al. 2014
**14**	*Elaphe quadrivirgata*	N/A	N/A	AB738958	AB738958	AB738958	Direct Submission
**15**		Ashiu, Kyoto, Kansai, Japan	As1352	N/A	N/A	HQ122007	Kuriyama et al. 2010
**16**	*Elaphe quatuorlineata*	Crkvino, Northern Macedonia	1509	MK334307	MK334307	MK334307	Jablonski et al. 2019
**17**		Galatas, Argolida, Greece	ZMUP 60	N/A	N/A	MH444348	Thanou et al. 2020
**18**	*Elaphe sauromates*	Taganrogskyi Gulf, Russia	ZISP 26197	N/A	MK640250	N/A	Jablonski et al. 2019
**19**		Solenoe Ozero, “Crimea”	1179	MK070315	MK070315	MK070315	Jablonski et al. 2019
**20**	*Elaphe schrenckii*	Changbai, Jilin, China	HS16031	MK193935	MK064638	MK201287	Li et al. 2020
**21**	*Elaphe taeniura*	Zhouzhi, Shaanxi, China	HS2010025	MK193982	MK064679	MK201334	Li et al. 2020
**22**		Heishiding, Guangdong, China	SYS r001057	MK194113	MK064790	MK201445	Li et al. 2020
**23**	*Elaphe urartica*	Kısıklı, Süphan Mts., Bitlis, Turkey	ZDEU 26/2012	N/A	MK640299	N/A	Jablonski et al. 2019
**24**		Guzdak, Qobustan, Azerbaijan	IZANAS T17	N/A	MK640269	N/A	Jablonski et al. 2019
**25**	*Elaphe zoigeensis*	Zoige, Sichuan, China	HS11251	MK193927	MK064630	MK201279	Li et al. 2020
**26**		Zoige, Sichuan, China	HS2010015	MK193930	MK064633	MK201282	Li et al. 2020
**27**	*Euprepiophis mandarinus*	HuangShan, Anhui, China	HS12062	MK193939	MK064643	MK201291	Li et al. 2020
**28**		HuangShan, Anhui, China	HS14017	MK193940	MK064644	MK201292	Li et al. 2020

DNA nucleotide sequences were aligned in the ClustalW algorithm with default parameters (Thompson et al. 1997) in MEGA 6 (Tamura et al. 2013). The aligned 16S, CO1 and cytb datasets were partitioned by codons with no gap positions allowed and applying default parameters in Gblocks version 0.91b (Castresana 2000). Three gene segments, with 1329 base pairs (bp) of 16S, 657 bp of CO1, and 585 bp of cytb, were concatenated seriatim into a 2571 bp sequence. With respect to the different evolutionary characters of each molecular marker, the dataset was split into seven partitions by gene and codon positions taking advantage of PartitionFinder 2.1.1 (Lanfear et al. 2012). The evolution models of each partition selected by PartitionFinder 2.1.1 were as follows: partition 1: 16S, GTR+I+G, 1287 bp; partition 2: COI\1, SYM+G, 219 bp; partition 3: COI\2, HKY+I, 219 bp; partition 4: COI\3, TVM+G, 219 bp; partition 5:;cytb\1, GTR+G, 195 bp; partition 6: cytb\2, HKY+I+G, 195 bp; partition 7:cytb\3, GTR+G 195 bp. General time-reversible (GTR) model. Sequence data were analyzed using Bayesian inference (BI) in MrBayes 3.2.4 (Ronquist et al. 2012), and maximum likelihood (ML) in RaxmlGUI 1.3 (Silvestro and Michalak 2012). Two independent runs were conducted in the BI analysis with 10,000,000 generations each and sampled every 1000 generations with the first 25% of samples discarded as burn-in, resulting in a potential scale reduction factor (PSRF) of < 0.005. In the ML analysis, a bootstrap consensus tree inferred from 1000 replicates was generated. Nodes with Bayesian posterior probabilities (BPP) ≥0.95 and ML support values of ≥70 were considered strongly supported (Huelsenbeck et al. 2001; Wilcox et al. 2002). Pairwise distances (*p*-distance) were calculated in MEGA6 using the uncorrected model. Gaps/Missing Data Treatment use the complete-deletion option, Substitutions to Include *d*: Transitions + Transversions option.

## Taxonomic accounts

### Elaphe xiphodonta sp. nov.

Taxon classificationAnimaliaSquamataColubridae

000ADEBF-FBFF-52E6-8FD0-2E7D9CB685ED

http://zoobank.org/AEA2D68D-5621-4B08-A4AE-BD99FA2192B9

#### Material examined.

***Holotype*.**SYS r002534, adult female (Figs 1, 2, 3A), collected by Yan-Bo Ma, Yi-Fei Gao on 4 September 2020 from Chengguan Town (33.58°N, 108.46°E (DD); ca 1731 m a.s.l.), Ningshaan County, Shaanxi Province, China. ***Paratypes*.**IVPP OV 2721, juvenile female (Fig. 3C), collected by Jing-Song Shi on 7 September 2020 from Chengguan Town (33.56°N, 108.50°E (DD); ca 1751 m a.s.l.), Ningshaan County, Shaanxi Province, China (Fig. 4).

**Figure 5.**
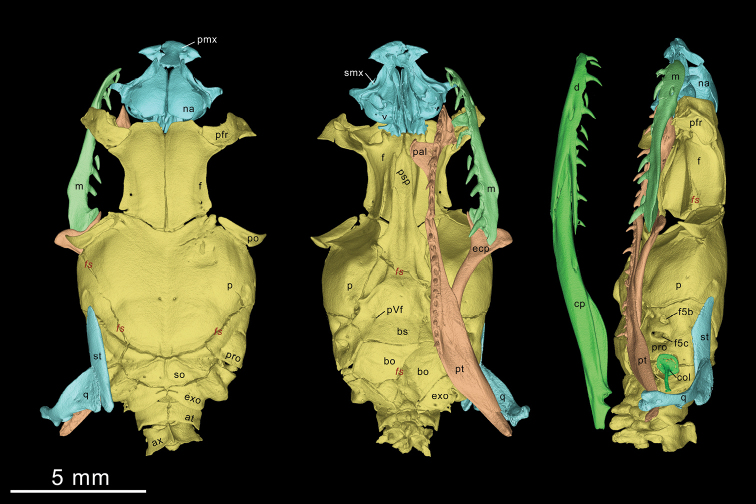
Three-dimensional model of the skull of *Elaphe xiphodonta* sp. nov. (IVPP OV 2721, paratype). Left, dorsal view; middle, ventral view; right, lateral view. (Right palatomaxillary arch, mandible and suspensorium are not shown). Implemented by Peng-Fei Yin, Ye-Mao Hou and Jing-Song Shi.

**Figure 6.**
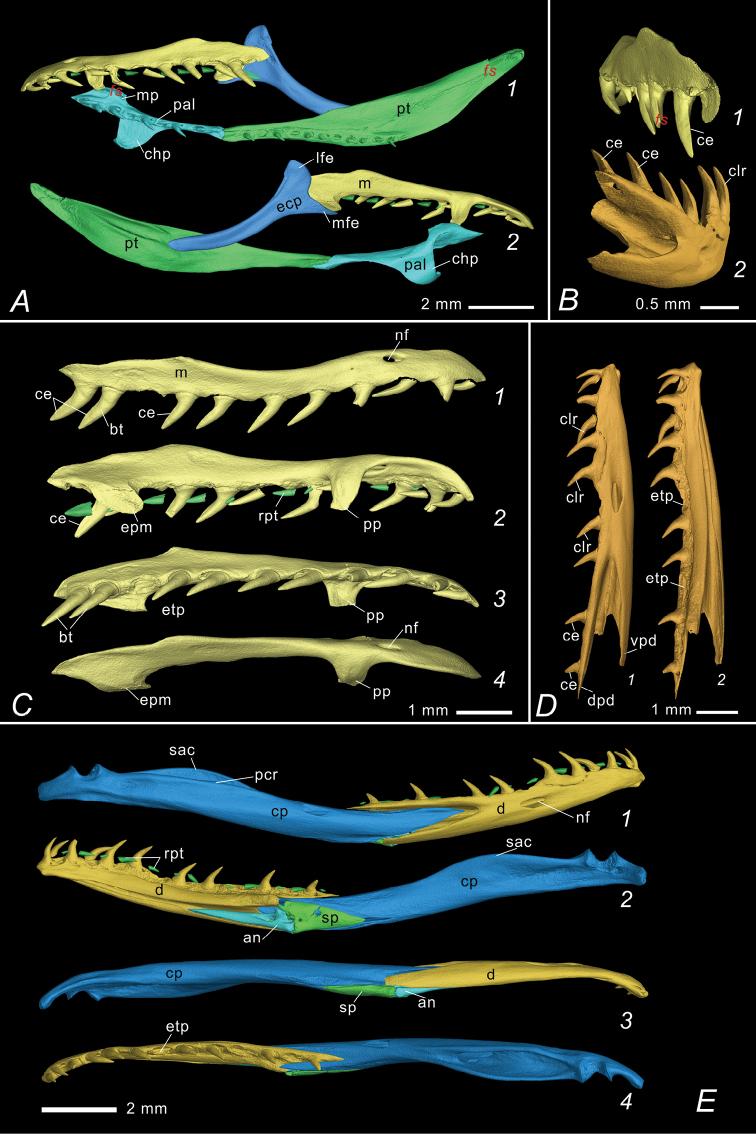
Palatomaxillary apparatus and mandibles of *Elaphe xiphodonta* sp. nov. (IVPP OV 2721, paratype). Implemented by Peng-Fei Yin, Ye-Mao Hou and Jing-Song Shi **A** ventral (A1) and dorsal (A2) view of left palatomaxillary apparatus **B** posterolateral view of left maxilla (B1) and right dentary (B2), with cutting edges (ce) and caudolateral ridges indicated **C** labial (C1, right), ventrolateral (C2, left), ventral (C3, right, mirrored) and dorsal (C4, left) view of maxilla **D** Labial (D1) and lingual (D2) view of right dentary **E** labial (E1), lingual (E2), ventral (E3) and dorsal (E4) view of right mandible. Abbreviations: an. angular, at. atlas, ax. axis, bo. basioccipital, bs. basisphenoid, bt. blade teeth (“xiphodont”), ce. Posterior cutting edge of the blade teeth, chp. choanal process of palatine, clr. caudolateral ridge of the teeth, cp. compound bone, d. dentary, dpd. dorsal process of dentary, ecp. ectopterygoid, epm. ectopterygoid process of maxilla, etp. empty tooth position, exo. exoccipital, f. frontal, f5b. foramen for maxillary branch of trigeminal, f5c. foramen for mandibular branch of trigeminal, lf. lacrimal foramen, lfe. lateral furcula of ectopterygoid, m. maxilla, na. nasal, mfe. mesial furcula of ectopterygoid, mp. maxillary process of palatine, nf, nutrient foramen, p. parietal, pcr. prearticular crest of compound bone, pfr. prefrontal, pmx. premaxilla, po. postorbital, pp. palatine process of maxilla, pro. prootic, psp. parasphenoid rostrum, pt. pterygoid, pVf. posterior Vidian foramen, rpt. replacement teeth, sac. surangular crest of compound bone, smx, septomaxilla, so. supraoccipital, sp. splenial, st. supratemporal, v. vomer, vpd. ventral process of dentary, *fs.* fracture surface.

**Etymology.** The specific epithet “*xiphodonta*” of the new species comes from the Ancient Greek “ξίφοσ (ksίfos, refer to ‘knife’ or ‘blade’)” and “δοντι (dónti, refer to ‘tooth’)”, meaning “blade-shaped teeth”, indicating that the new species has unique blade-shaped MT and DT (Figs 5, 6), which differs from the inconspicuous dental specializations (all teeth are cone-shaped) in its congeners. We suggest the Chinese formal name as “秦皇锦蛇” (Qín Huáng Jǐn Shé), which derived from Qin Shi Huang (personal name: Ying Zheng or Zhao Zheng; 259 BC–210 BC), the founder of the Qin dynasty and the first emperor of unified China, whose territory including the distribution range of *Elaphe xiphodonta* sp. nov. The English name is suggested as “Qin Emperor Rat Snake” or “Blade-teethed Rat Snake”.

#### Diagnosis.

*Elaphe xiphodonta* sp. nov. can be differentiated from its congeners by the combination of the following morphological characters: (1) medium body size , SVL 785 mm in single adult female; (2) dorsal scales in 21-21-17 rows, the medial 11 rows keeled; (3) supralabials seven or eight, third/fourth (right) or fourth/fifth (left) in contact with eye, infralabials 9 or 10; (4) ventral scales 202–204; (5) subcaudals 67–68; (6) loreal single, not in contact with eye, not in contact with internasals; (7) two preoculars (including one subpreocular), two postoculars; (8) two anterior temporals, three posterior temporals; (9) precloacal plate divided; (10) reduced teeth number in maxilla and dentary bones (MT 9+2, DT 12; (11) sharp edges on the posterior or posterolateral surface of the rear MT and DT; (12) top of head yellow, three distinct markings on head and neck; (13) a distinct black labial spot present on supralabials; (14) ground color of dorsum yellow, 46–49 entire (or incomplete) reddish brown blotches with black edges on body and 12–19 similarly colored spots on tail; (15) ventral surface of body yellow with mottled irregular black blotches, a few irregular small red spots dispersed on middle of ventral scales.

#### Description of holotype.

Adult female (Figs 1, 2, 3A). Body and tail slender, TL 967.5 mm (SVL 785.2 mm, TaL 182.3 mm, TaL/TL: 0.19); dorsal scales in 21–21–17 rows, the medial 11 rows keeled, the vertebral scales not enlarged; head elongate, moderately distinct from neck, longer than width and narrow anteriorly, HL 26.5 mm, HW 18.1 mm (HW/HL: 0.68); eye medium, ED 3.2 mm, pupil elliptic; rostral triangular, broader than high, RW 7.1 mm, RH 4.5 mm (RW/RH: 1.58; RW/HW: 0.39), visible from dorsum; nostril laterally pointed, located in the middle of nasal; nasal divided into two scales by nostril; two internasals, anteriorly rounded, bordered by two large prefrontals posteriorly; frontal single and enlarged, narrowed posteriorly; parietals paired, longer than width, in contact with each other medially, with upper anterior and posterior temporals laterally; one loreal on each side, in contact with nasal anteriorly, preocular posteriorly, prefrontals dorsally and the second supralabial ventrally; one enlarged preocular in contact with eye and supraocular posteriorly, prefrontal and loreal anteriorly, a smaller subpreocular ventrally, and not in contact with frontal; subpreocular in contact with eye and the third supralabial posteriorly, the second supralabial anteriorly, and preocular dorsally; two postoculars, upper one in contact with eye anteriorly, supraocular and parietal dorsally, and upper anterior temporal posteriorly, bottom one in contact with eye anteriorly, with upper and lower anterior temporals posteriorly, fifth and sixth supralabials below on left, and with fourth and fifth supralabials below on right; eight supralabials on left, seven supralabials on right (the third and the forth merged relative to left), first and second in contact with nasal, fourth and fifth reaching orbit on left, third and fourth reaching orbit on right; ten infralabials on left, nine infralabials on right, first pair in broad contact with each other, first to fifth in contact with anterior pair of chin shields, fifth in contact with posterior chin shields, fifth infralabial bipartitioned, lower part obviously larger than upper one; two pairs of chin shields, elongate, anterior pair larger, first pair meeting in midline, posterior pair is separated by three small scales; two similarly sized anterior temporals on left, lower one divided into two small scales on the right; three similarly sized posterior temporals on each side; 204 ventrals, excluding two preventrals; 67 pairs of subcaudals, excluding tail tip; precloacal plate divided.

**Table 3.** Uncorrected *P*-distance of CO1 gene among 17 *Elaphe* species used in this study.

		1	2	3	4	5	6	7	8	9	10	11	12	13	14	15	16	17
1	*Elaphe xiphodonta* sp. nov.	0.0																
2	*E. anomala*	12.2	/															
3	*E. bimaculata*	16.4	12.9	/														
4	*E. cantoris*	14.2	17.9	18.4	/													
5	*E. carinata*	13.9	11.3	13.4	17.9	0.3												
6	*E. climacophora*	15.7	16.0	15.7	19.1	14.4	0.0											
7	*E. davidi*	14.4	12.2	11.5	19.5	13.5	13.5	/										
8	*E. dione*	15.3	16.0	10.9	18.2	14.3	13.9	14.2	/									
9	*E. hodgsonii*	13.8	17.1	16.7	13.7	16.2	17.3	14.8	16.4	/								
10	*E. moellendorffi*	14.6	16.4	17.1	13.7	16.6	16.1	17.4	16.8	12.2	/							
11	*E. quadrivirgata*	10.7	8.7	12.5	17.9	8.5	14.9	11.4	12.6	13.4	16.6	/						
12	*E. quatuorlineata*	17.1	14.1	12.5	18.4	13.8	17.9	12.5	13.6	16.1	15.8	12.5	/					
13	*E. sauromates*	15.5	13.9	14.1	19.1	15.5	14.7	14.5	13.4	17.3	16.6	15.6	8.5	0.3				
14	*E. schrenckii*	12.2	0.0	12.9	17.9	11.3	16.0	12.2	16.0	17.1	16.4	8.7	14.1	13.9	/			
15	*E. taeniura*	15.2	16.3	16.7	16.0	16.0	15.5	15.6	15.3	13.2	16.2	13.8	14.7	16.1	16.3	6.8		
16	*E. urartica*	14.1	14.0	13.9	16.8	12.7	13.7	13.3	11.8	15.2	15.6	12.5	9.4	8.9	14.0	16.5	0.0	
17	*E. zoigeensis*	8.4	13.3	14.9	16.9	15.0	16.4	13.3	15.5	13.5	14.6	12.3	16.0	14.2	13.3	16.3	14.0	0.0

**Table 4.** Uncorrected *P*-distance of cytb gene among 16 *Elaphe* species used in this study.

		1	2	3	4	5	6	7	8	9	10	11	12	13	14	15	16
1	*Elaphe xiphodonta* sp. nov.	0.0															
2	*E. anomala*	17.1	/														
3	*E. bimaculata*	13.7	17.0	/													
4	*E. cantoris*	17.2	16.6	18.2	/												
5	*E. carinata*	18.7	14.1	14.6	19.1	0.0											
6	*E. climacophora*	17.9	11.0	16.2	19.4	12.9	0.4										
7	*E. davidi*	18.8	15.2	12.1	17.2	14.8	15.9	/									
8	*E. dione*	19.6	19.0	15.0	15.2	18.6	17.6	16.1	/								
9	*E. hodgsonii*	16.7	15.5	17.1	14.5	17.7	15.0	14.1	19.6	/							
10	*E. moellendorffi*	21.7	18.5	21.8	16.3	16.4	17.6	18.6	20.7	14.8	/						
11	*E. quadrivirgata*	19.7	13.1	15.4	16.9	12.6	13.3	13.8	15.5	15.8	18.9	0.9					
12	*E. quatuorlineata*	16.7	13.0	16.5	14.5	13.7	11.0	13.7	15.5	17.4	17.1	11.7	0.0				
13	*E. sauromates*	20.0	15.3	18.6	14.3	16.0	15.5	15.4	20.8	17.2	17.5	15.6	6.8	/			
14	*E. schrenckii*	17.7	0.4	17.6	17.2	14.7	11.6	15.2	18.3	16.1	19.1	12.5	13.5	15.9	/		
15	*E. taeniura*	16.3	17.8	15.4	12.8	16.1	17.1	15.0	16.6	12.9	15.9	14.7	14.0	15.0	18.4	5.4	
16	*E. zoigeensis*	13.3	16.3	16.4	17.1	20.4	16.5	18.3	15.4	12.5	20.0	16.6	14.3	16.0	16.9	15.7	0.0

#### Coloration in life.

Dorsal surface of head yellow, three distinct markings on head and neck; the anterior transverse black stripe, somewhat reddish medially, extends from the posterior margin of rostral and through the each eye to last two supralabials and adjacent small scales; interorbital arcuate cross-band, covering anterior part of frontals, most part of prefrontals, top of supraoculars, bottom half of upper postoculars, intact bottom postoculars, bottom half of upper temporals, most of bottom of temporals, dorsal edge of sixth left supralabials (fifth on the right), dorsal half of seventh supralabials (sixth) and bottom half of posterior-most supralabials, not reaching internasals, connected to largest posterior marking from mediolateral part; largest marking is a distinct black “M”-shaped marking that is reddish medially, covering the posteromedial part of the frontals, posterior part of supraoculars, most of parietals, dorsal margin of upper temporals, posteriorly extended, forming two thick black-edged reddish brown stripes on nape. Lateral surface of head yellow, a few small black spots dispersed on supralabials (2, 4 and 5 on left and 2, 3 and 4 on right) and subpreocular, a distinct scutellate black labial spot on the junction of the 2, 3 and right subpreocular (absent on left). Ventral surface of head consistently light-yellow, a few small black spots dispersed on the mental, infralabials, and anterior chin shields. An irregular spot occurs on the posterior edge of the junction of two anterior chin shields. Mouth lining is pale-heather and tongue is black.

Ground color of dorsal surface yellow, 49 complete or incomplete, black-edged reddish brown blotches on body and 12 similarly colored spots on tail; dorsal blotches on body approximately three to five scales in length, and eight to eleven scales rows in width; each blotch is usually composed of reddish brown scale with dark-brown edges. Two rows of smaller, black-edged reddish brown blotches on both side of the larger mid-body blotches, alternating with the mid-body blotches, each blotch covers 2–4 dorsal scales and separated from ventral scales by two rows of the dorsolateral scales. Ground color of ventral surface is yellow, mottled with irregular black blotches, a few irregular small red spots scattered midventrally.

#### Intraspecific variation.

Measurements, body proportions and scale and pattern counts of the two specimens are listed in Table 5. The third and fourth supralabials are merged on right in holotype, which leads to the different supralabial counts. Regardless of the slight variation in scalation among the type series of *E. xiphodonta* sp. nov., the color pattern varies considerably between the juvenile and the adult: In adult one (SYS r002534, holotype), the color of the rostral, top and side of head, and chin, as well as dorsal and ventral sections of body are consistently light-yellow, whereas in the juvenile (IVPP OV 2721), the color of head and dorsal body is greyish to olive-green; the ventral scales and subcaudal scales are oyster white, with irregular greyish black spots. The juvenile has a similar dorsal pattern as the adult.

**Table 5.** Measurements and scale counts and body proportions of *Elaphe xiphodonta* sp. nov.

Voucher	SYS r002534	IVPP OV 2721
**Sex**	female	female
**SVL**	785.2	307.5
**TaL**	182.3	62.5
**TL**	967.5	370.1
**TaL/TL**	0.19	0.17
**HL**	26.5	17.64
**HW**	18.1	11.14
**HW/HL**	0.68	0.63
**ED**	3.2	2.8
**RW**	7.1	3.4
**RH**	4.5	2.0
**RW/RH**	1.58	1.70
**RW/HW**	0.39	0.31
**DSR**	21-21-17	21-21-17
**SPL**	8/7	8/8
**IFL**	10/9	11/11
**CS**	4 (2 pairs)	4 (2 pairs)
**V**	204	202
**SC**	67	67/68
**MT**	11 (9+2)	11 (9+2)
**Dorsal blotches**	61 (49+12)	65(46+19)

#### Skull.

The osteological description is based on a road-killed juvenile individual (Paratype, IVPP OV 2721, Figs 5, 6). The skull is nearly completely preserved except for the slightly crushed parietal, and basioccipital bones.

**Snout (Fig. 5).** The premaxilla is short and blunt. The ascending process is laterally expanded and slanted posteriorly as in many borrowers (e.g., *Euprepiophis* and *Archelaphe*), rendering it hourglass-shaped in anterior view. The transverse process of premaxilla is flat, triangular-shaped in dorsal view. The posterior end of vomerine process of premaxilla contacts the tip of both vomer and septomaxilla. The posteromedial surface of vomerine process is expanded and oval shaped. The anterior section of the dorsal plate of nasal is tapered while the posterior section is expanded as oval shaped. The dorsal surface is slightly bulged.

**Braincase (Fig. 5).** The parietal is blunt and rounded, lacks a lateral crest on each side. The prefrontal is slender, somewhat rectangular. The anterolateral process is blunt. The anterior margin of frontal presents as trapezium-shaped. The lateral margin slightly concave. The postorbital is crescent-shaped, the anterodorsal process does not contact the frontal. The ventral process of postorbital is tapered, not furcated. The supraoccipital is rectangular and compressed, bearing a trifurcated dorsal ridge. The basisphenoid is wide and flat, with no conspicuous ridges on the ventral surface. The rostrum of parasphenoid is not divided. The angular surface between the basisphenoid and basioccipital is smooth. The columella is stubby, the shaft of columella quite short, approximately 1.2 times as length as the diameter of foot plate. The foramina for maxillary branch of trigeminal (f5b) and mandibular branch of trigeminal (f5c) nerves are oval shaped, not reaching the margin of prootic. The f5b is approximately 1/2 the width of f5c.

#### Palatomaxillary arch

**(Fig. 6)** The maxilla is slender. The maxillary nutrient foramen is transversely elongate, oval shaped and opens on the anterolateral side at the level of the third maxillary tooth and perforates the maxilla laterally. The anterior portion of maxilla curves slightly inward. The palatine process of maxilla is elongate while the ectopterygoid process is short and bunt.

The maxilla has 11 teeth on each side, with one or two rows of replacement teeth on the lingual side. In contrast to other species of *Elape*, the maxillary dentition of the new species is conspicuously differentiated. The anterior five MT have inconspicuous posterolateral ridges, while the posterior six teeth have a sharply edged ridge on their posterior margin (which could be also described as: the posterolateral ridge gradually moved posteriorly by the MT row, forming a sharp cutting edge on the posterior MT), forming blade-like teeth. The MT increase in size posteriorly, the posterior-most two being the largest, not separated from the anterior teeth by a diastema. The cutting edges of the posterior four MT slightly posteriorly convex, rendering them kukri shaped.

The palatine bears nine teeth, with one row of replacement teeth on the labial side. The choanal process of palatine (chp) forms a right triangular in dorsal view. The anterolateral margin of the maxillary process forms an approximate 30° angle with the medial line. The posterior margin of maxillary process is perpendicular to the medial line and collinear with the anterior margin of the choanal process. The pterygoid is slender and lanceolate in shape, 1.8 times the length of palatine, bearing 12 solid teeth (with one row of replacement teeth on the labial side). The ectopterygoid process and the medial transverse process of pterygoid are very small and difficult to see. The ectopterygoid is horizontally expanded, and outwardly curved in dorsal view. The labial furcula of ectopterygoid is oval, and distinct from the lingual process. The medial furcula (medial process) is elongate and spiculate, slightly curved ventrally.

#### Suspensorium and mandible

**(Figs 5, 6)** The mandible is slender and moderately curved. The supratemporal is flat and slender, fusiform, and the anteroventral margin is slightly angulated upward. The quadrate is triangulated in lateral view, and approximately the same length as supratemporal. The supratemporal angular surface of the quadrate is expanded, elongated and oval shaped. The prearticular crest (pcr) of the compound bone is prominent while the surangular crest (sac) is absent. The angular is slender and triangular. The splenial is triangular, coracoid shaped and shorter than angular. The dental bone bears 13 teeth, decreasing in size at the fifth tooth, with one or two rows of replacement teeth on the lingual side. The posterior tip of dorsal process of dentary extends farther posteriorly than the ventral process. The largest dentary nutrient foramen is elongated-oval shaped and lies below the seventh tooth.

#### Dentition

**(IVPP OV 2721, paratype)** Maxilla: 11/11 (9+2); pterygoid: 12/12; palatine: 9/9; dentary: 13/13. Dentitional comparisons within the genus *Elaphe* and some related colubrid groups are listed in Table 6.

**Table 6.** Dentition comparison of the *Elaphe* species and related colubrid species.

Taxon (*n*)	Maxillary	Blade teeth	Palatine	Pterygoid	Dentary	Reference
*** Elaphe ***						
*E. xiphodonta* sp. nov. (1)	11/11	Y	9/9	12/12	13/13	this study
*E. bimaculata* (1)	19/20	N	9/10	15/16	18/19	this study
*E. carinata* (5)	17	N	9–11	13–16	19–21	this study
*E. climacophora* (2)	17	N	11	17–20	23–25	Helfenberger and Schätti 1998
*E. dione* (2)	16–20	N	7–9	12–13	18–23	this study
*E. davidi* (3)	16–17	N	9–12	12–16	16–19	Helfenberger and Schätti 1998
*E. moellendorffi* (2)	22–23	N	11	19–22	27–29	this study
*E. schrenskii* (2)	16–17	N	10–11	12–13	19–21	this study
*E. taeniura* (3)	17–23	N	10–11	16–19	19–23	this study
*E. zoigeensis* (2)	14–17	Y	10–10	10–13	16–17	this study
*** Euprepiophis ***						
*Eu. mandarinus* (2)	16–19	Y	10–11	19–24	19–22	this study
*Eu. perlaceus* (2)	20–20	Y	12–12	18–21	21–22	this study
*** Coelognathus ***						
*C. flavolineatus* (1)	23	N	11/13	25/26	27/28	this study
*C. philippinus* (1)	25	N	12/13	26/27	29/28	this study
*C. radiatus* (5)	20–23	Y	10–12	18–24	23–27	this study
*** Oligodon ***						
*Ol. ornatus* (1)	8/8	Y	4/4	5/5	13/13	this study
*** Oocatochus ***						
*Oo. rufodorsatus* (2)	18–19	Y	10–11	17–18	21–23	this study
*** Gonyosoma ***						
*G. oxycephalum* (1)	23/23	N	10/10	14/13	26/26	this study
*** Ptyas ***						
*P. carinata* (2)	24–25	Y	17–15	19–20	22	this study
*P. dhumnades* (1)	26	Y	21/20	23	26	this study
*** Dasypeltis ***						
*D. scabra* (2)	7–6	N	8–9	0	3–3	Helfenberger and Schätti 1998
*** Thermophis ***						
*T. zhaoermii* (2)	19–21	Y	12–16	18–23	21/24	this study

Abbreviations: *n.* number of referenced specimens (if *N* = 1, the dentition count are listed as: left/right; *N* > 1: minimum – maximum).

#### Comparisons.

**Figure 7.**
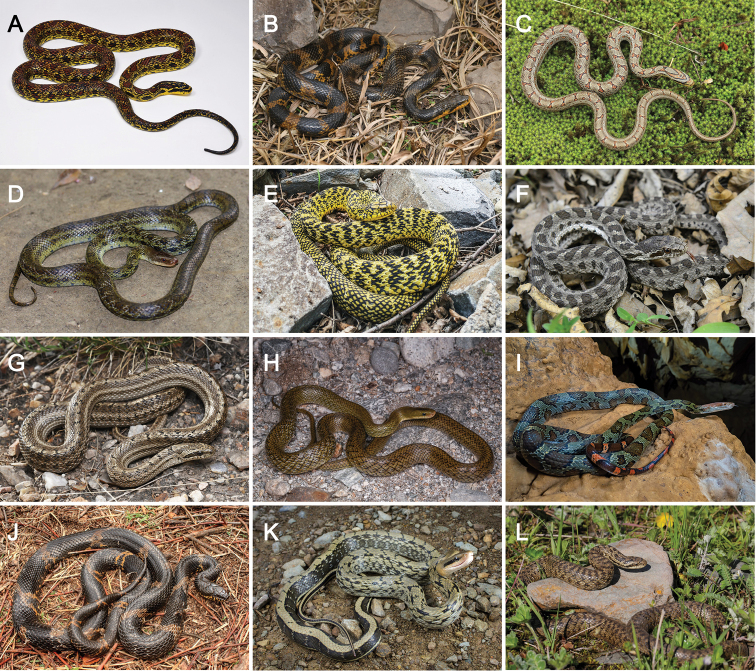
Comparisons of general morphological characteristics with its congeners in China **A***Elaphe xiphodonta* sp. nov. (SYS r002534, holotype), Ningshaan, Shaanxi, by Shuo Qi **B***E. anomala*, Benxi, Liaoning, by Shuo Qi **C***E. bimaculata*, Hong’an, Hubei, by Chong-Jian Zhou **D***E. cantoris*, Bomê, Tibet, by Jing-Song Shi **E***E. carinata*, Mentougou, Beijing, by Jing-Song Shi **F***E. davidi*, Benxi, Liaoning, by Jing-Song Shi **G***E. dione*, Yongdeng, Gansu, by Shuo Qi **H***E. hodgsonii*, Gyirong, Tibet, by Shuo Qi **I***E. moellendorffi*, Chongzuo, Guangxi, by Jia-Jun Zhou **J***E. schrenckii*, Baishan, Jilin, by Shuo Qi **K***E. taeniura* from Hangzhou, Zhejiang, by Wei-Liang Xie **L***E. zoigeensis*, Jiuzhaigou, Sichuan, by Jin-Wang.

Detailed comparisons among *Elaphe* species are given in Table 7 and Fig. 7.

*Elaphe xiphodonta* sp. nov. is distinct from all of its congeners by having fewer MT, enlarged posterior MT, cutting edges on both MT and DT, and three rows of large, black-bordered reddish brown dorsal blotches.

Additionally, *Elaphe xiphodonta* sp. nov. can be distinguished from *E. cantoris*, *E. climacophora* (Boie, 1826), *E. hodgsoni*, *E. moellendorffi*, and *E. taeniura* by having fewer ventral scales (202–204 vs. 226–239 in *E. cantoris*, 222–236 in *E. climacophora*, 228–247 in *E. hodgsoni*, 270–278 in *E. moellendorffi*, and 223–261 in *E. taeniura*), fewer subcaudals (67–68 vs. 78–87 in *E. cantoris*, 97–116 in *E. climacophora*, 72–92 in *E. hodgsoni*, 92–102 in *E. moellendorffi*, and 73–121 in *E. taeniura*), smaller body size (SVL 785 mm in single adult female vs. maximum SVL 1158 mm in *E. cantoris*, > 2000 mm in *E. climacophora*, 1190 mm in *E. hodgsoni*, 1602 mm in *E. moellendorffi*, and > 2000 mm in *E. taeniura*), and vastly different color pattern (Table 7).

**Table 7.** Diagnostic characters separating all 17 species of the *Elaphe*, with distinguishing characters marked in bold. *: Counts of PrO contain subpreocular.

Species	maximum SVL (in mm)	DSR	SPL	IFL	PrO*	PtO	TMP	V	SC
*Elaphe xiphodonta* sp. nov.	785	21-21-17	8 (7)	9 (10)	2	2	2+3	202–204	67–68
*Elaphe anomala*	**1925**	23 (21–25)-23 (19–23)-19 (17–19)	8	9–11	2 (1)	2 (1)	2 (3)+3 (2)	203–225	45–77
*Elaphe bimaculata*	760	**23 (23–25)-23 (21–25)-19 (21)**	8 (7)	9–12	2 (1)	2	2+3	170–209	61–81
*Elaphe cantoris*	**1158**	19 (20, 21)-19 (21–23)-17	8	9–10	2	2	2+3 (2)	**226–239**	**78–87**
*Elaphe carinata*	**> 2000**	23 (21–25)-23 (21–25)-19 (17)	8 (9)	9–12	2 (1)	2	2+3	186–227	**69–102**
*Elaphe climacophora*	**> 1500**	**NA-23 (25)-NA**	8 (9)	11	2	2	2+3 (2)	**222–236**	**97–116**
*Elaphe davidi*	**1227**	**25 (22–27)-23 (22–25)-19 (17–21)**	8 (7)	**11–13**	2 (1, 3)	2 (1–4)	2 (1, 3)+4 (2–3)	**155–183**	53–72
*Elaphe dione*	893	25 (21–27)-25 (21–27)-19 (17–21)	8 (9)	9–11	2 (1)	2	2+3	168–206	51–84
*Elaphe hodgsoni*	**1190**	23 (21–25)- 23 (21–25)-17	8 (9)	9–12	2 (1)	2	2 (1, 3)+3 (2, 4)	**228–247**	**72–92**
*Elaphe moellendorffi*	**1602**	**25 (23–27)-27 (5)-19 (21)**	**9 (10)**	10–13	2	2	2 (3)+3 (4)	**270–278**	**92–102**
*Elaphe quadrivirgata*	**>1000**	**NA-19-NA**	8	**11**	2	2	2 (1)+3 (2)	195–215	**70–96**
*Elaphe quatuorlineata*	**> 2000**	**25-25 (23–27)-19**	8 (9)	11	2 (3)	2 (3)	2 (3)+3 (4)	187–234	56–90
*Elaphe sauromates*	**1250**	**25 (21–27)-25 (23, 24)-19 (18–21)**	8 (7–10)	11 (9–12)	2 (1, 3)	2 (1)	2(1, 3)+ 4 (2–5)	199–222	61–79
*Elaphe schrenckii*	**1335**	**23 (21)-23 (21)-19**	8 (7)	8–11	2 (1)	2	2+3 (2)	**208–224**	57–75
*Elaphe taeniura*	**> 2000**	**25 (23)-23 (21, 25)-19 (17)**	9 (6–10)	9–13	2 (1)	2 (3)	2 (1, 3)+3 (2–5)	**223–261**	**73–121**
*Elaphe urartica*	970	**25 (23, 24)- 25 (23, 24)-19 (18)**	8 (9)	11 (10–13)	2 (1, 3)	2 (1, 3)	2 (3)+ 4 (2, 3)	154–211	60–74
*Elaphe zoigeensis*	722	21-19(21)-17	7–8	9	**3**	2	2+3(2)	202–212	68–79

*Elaphe xiphodonta* sp. nov. can be differentiated from *E. quatuorlineata* Lacepede, 1789, *E. sauromates* (Pallas, 1811) and *E. urartica* (Jablonski, Kukushkin, Avci, Bunyatova, Ilgaz, Tuniyev & Jandzik, 2019) by having fewer dorsal scale rows (21-21-17 vs. 25-25 (23–27)-19 in *E. quatuorlineata*, 25 (21–27)-25 (23, 24)-19 (18–21) in *E. sauromates* and 25 (23, 24)- 25 (23, 24)-19 (18) in *E. urartica*) and a vastly different color pattern. Beyond that, *E. xiphodonta* sp. nov. can be further differentiated from *E. quatuorlineata* and *E. sauromates* by its smaller body size (SVL 785 mm in single adult female vs. maximum SVL > 2000 mm in *E. quatuorlineata* and 1250 mm in *E. sauromates*).

*Elaphe xiphodonta* sp. nov. can be differentiated from *Elaphe carinata*, *E. davidi* and *E. quadrivirgata* (Boie, 1826) by its smaller body size (SVL 785 mm in single adult female vs. maximum SVL > 2000 mm in *E. carinata*, 1227 mm in *E. davidi*, and > 1000 mm in *E. quadrivirgata*), having fewer subcaudals (67–68 vs. 69–102 in *E. carinata*, 70–96 in *E. quadrivirgata*), and a vastly different color pattern.

Despite the morphological similarities to *E. bimaculata*, *E. dione*, and *E. zoigeensis*, *Elaphe xiphodonta* sp. nov. differs from them by having different dorsal scale row counts (21-21-17 vs. 23 (23–25)-23 (21–25)-19 (21) in *E. bimaculata*), fewer preoculars (2 vs. 3 in *E. zoigeensis*), fewer MT (11/11 vs. 19/20 in *E. bimaculata*, 16–20 in *E. dione*, and 14–17 in *E. zoigeensis* (Table 6), blade-like posterior MT and the unique color pattern within the genus *Elaphe*.

#### Molecular phylogeny.

**Figure 8.**
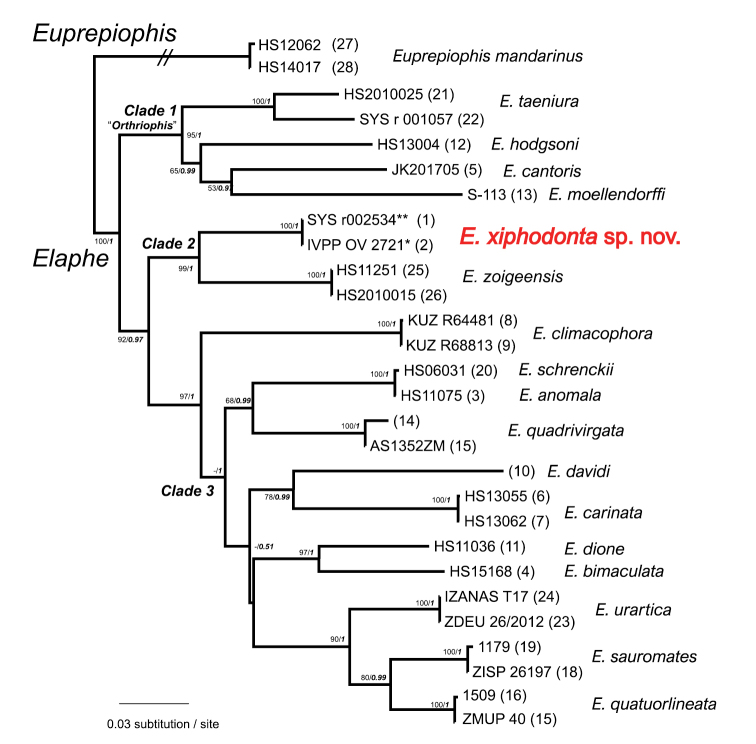
Bayesian inferenced topology of 13 *Elaphe* species, based on the four partial mitochondrial DNA sequences (12S rRNA, 16S rRNA, CO1 and Cytb genes). BPP and BS values, respectively, occur at the nodes.

The ML and BI analyses produced identical topologies, which were integrated in Fig. 8. The pairwise distances based on CO1 and cytb genes among all species are given in the Tables 3, 4, respectively.

The phylogenetic analyses recovered a strongly supported monophyletic lineage containing all *Elaphe* (BS 100; BPP 1.00) which can be divided into three strongly supported clades (BS 95–97; BPP 1.00). Clade 1 includes all species previously in the genus “*Orthriophis*” with strong nodal support (BS 95; BPP 1.00). Notable intraspecific genetic differentiation within *E. taeniura* (mean *p*-distances 6.8% in CO1, 5.4% in cytb), pertains to different geographical clades.

The two samples from Chengguan Town, Shaanxi are clustered together with strong support (BS 100; BPP 1.00) with nearly no molecular divergence (mean *p*-distances 0% in CO1, 0% in cytb) between them. The clade of the above-mentioned specimens constitutes a sister clade with *E. zoigeensis* (Clade2, mean *p*-distances 8.4% in CO1, 13.3% in cytb).

Within Clade 3, the relationship between *Elaphe anomala* and *E. schrenckii* are worth noting. These two species form a strongly supported monophyletic group (BS 100; BPP 1.00) bearing low interspecific molecular divergence (mean *p*-distances 0.0% in CO1, 0.4% in cytb), suggesting they may be different color morphs of the same species, as mention before (An et al. 2010). However, given their distinctive color pattern differences and obvious geographical separation, we follow the current taxonomy.

Based on their phylogenetic relationships, genetic differentiation, and morphological distinctiveness (see Taxonomic accounts below), we hypothesize that the population from Chengguan Town, Shaanxi represents a separately evolving lineage and should be described as a new species.

#### Distribution, ecology and habit.

**Figure 9.**
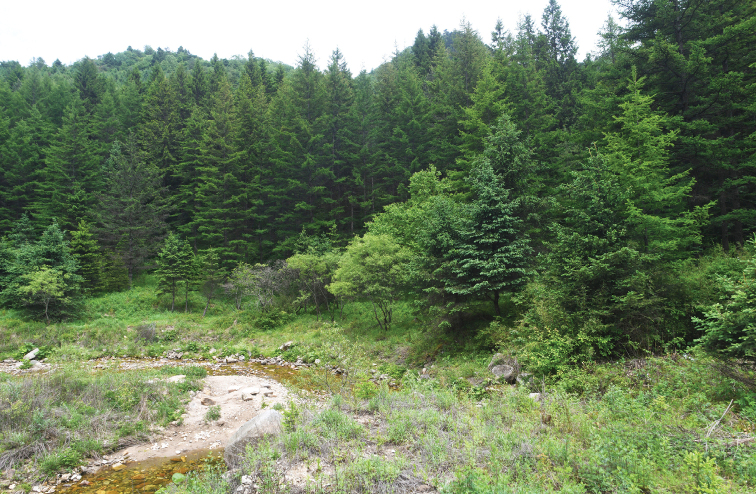
Habitats of *Elaphe xiphodonta* sp. nov. (Ningshaan County, Shaanxi), Provide by Shu-Hai Bu.

*Elaphe xiphodonta* sp. nov. is currently known only from the Chengguan Town, Ningshaan County, Shaanxi Province, China. The new species inhabits sunny or semi-sunny gravels and bushes on slopes of less than 20°, along Chang’an River with an average width of 3 m. Elevation of the habitat ranges from 1700 to 1900 m. The vegetation types are *Abies fargesii* forest with artificial *Picea asperata*, *Salix fargesii*, *Rubus koereana*, *Betula albosinensis* and *Fargesia qinlingensis*. The canopy density is 0.75 (Bu and Zheng 2015). The new species is sympatric with *Nanorana unculuanus*, *Scutiger ningshanensis*, *Euprepiophis perlaceus*, *Rhabdophis nuchalis*, *Stichophanes ningshaanensis*, *Gloydius qinlingensis* and *Protobothrops jerdonii* (Fig. 9). When being captured, the new species flattens its head triangular and releases scent from the cloacal scent glands with smells a bit similar to *P. jerdonii*.

In feeding habits, the fecal samples from the holotype were checked and contained only feathers, indicating that this species is at least a bird-eater. The holotype was observed to prey on captive nesting quail and quail egg.

## Discussion

This study described a new *Elaphe* species which has a unique coloration and specialized teeth and had been overlooked for a long time during previous surveys. The new species was mistaken for *P. jerdonii*, which it mimics, at first glance during the field work. Nevertheless, the mimicry in *E. xiphodonta* is not unique within *Elaphe*. The coloration and head shape of *E. davidi* mimics that of the sympatric *Gloydius* spp. (Zhao 2006). *Gloydius qinlingensis* and *Protobothrops jerdonii* are sympatric with *E. xiphodonta*. *Elaphe xiphodonta* is similar to *P. jerdonii* in body shape, coloration, pattern and cloacal gland odor. Therefore, we hypothesize that *E. xiphodonta* is mimicking the model *P. jerdonii* as a means to avoid predation. Additional observations concerning the potential mimic-model relationship between *E. xiphodonta* and *P. jerdonii* are necessary.

*Elaphe dione* was previously widely recorded from northern China (Zhao 2006). Its distribution is currently restricted to Qinling Mountains-Huaihe River Line, and the records from other localities may belong to different taxa (e.g., *E. zoigeensis*, Huang et al. 2012). Previously collected specimens designated as *E. dione* (e.g., Fang and Song 1981) from Ningshaan County and surrounding areas are worth carefully re-examining.

Based on the dentition comparison in Table 6, it is clear that *E. xiphodonta* has far fewer MT and DT than most other species of *Elaphe*, which have more than 14 MT and DT. The tooth morphology of *E. xiphodonta* is similar to that of the genus *Oligodon* in a number of ways. Their reduced number and the specialized shape might relate to their unusual diet, since the morphology of the teeth in snakes have yet to be correlated with diet and prey-handling behaviors (Cundall and Irish 2008; Shi 2020). Some lizard-eating snakes tend to have numerous and closely spaced teeth (e.g., *Scaphiodontophis*, *Sibynophis* and *Liophidium*Zaher et al., 2012) while many oophagous snakes have reduced numbers of MT and DT (e.g., *Dasypeltis*). Blade-like rear teeth (sickle or kukri in shape, with sharp cutting edges on the posterior margins of two or more posterior MT) occur widely in many clades of the superfamily Colubroidea (e.g., Colubridae, Dipsadidae, Natricidae and Pseudoxenodontidae), most of which are anuran predators (e.g., *Heterodon*, *Hydrodynastes* and *Rhabdophis*) while some of the others tend to feed on eggs (e.g., *Oligodon*, *Prosymna* and *Cemophora*) or lizards (e.g., *Orientocoluber* and *Hemorrhois*). The diet and prey behaviors of the above-mentioned snakes have been widely reported in previous studies (Henderson 1984; Cundall and Greene 2000; Cundall and Irish 2008; Zaher et al. 2012), whereas far fewer comprehensive studies have focused on the function of blade-like teeth. A recent study indicated that a large proportion of anuran predators have blade-like rear teeth. Some snakes have been observed to deflate their anuran prey by puncturing and slicing their skin (e.g., *Pseudoagkistrodon rudis*; Shi 2020) or to consume the egg liquid (or embryos) of reptile eggs by cutting through their shells (e.g., *Oligodon*, *Prosymna* and *Cemophora*). Hence, we speculate that *E. xiphodonta* preys on frogs or eggs in the wild. In view of the limited numbers of the specimens in the present study, further field observation is needed to verify the above-mentioned hypothesis.

## Supplementary Material

XML Treatment for Elaphe xiphodonta

## Appendix 1

Details of the osteological specimens examined for dentition comparisons in this study.

**
Colubridae
**

*
Elaphe
*

*E. xiphodonta* sp. nov. IVPP OV 2721, 3D model of impregnated specimens, IVPP.

*E. zoigeensis*IVPP OV 2672, 3D model of impregnated specimens, IVPP.

*E. carinata*IVPP OV 2296, osteological specimens, IVPP.

*E. climacophora* SH 530, lateral and dorsal views of skull (Helfenberger and Schätti 1998).

*E. davidi*BM 1916.1.15.17, lateral and dorsal views of skull, BM (Helfenberger and Schätti 1998).

*E. dione*IVPP OV 2302, osteological specimen, IVPP.

*E. moellendorffi*IVPP OV 2686, osteological specimen, IVPP.

*E. schrenskii*IVPP OV 2295, osteological specimen, IVPP.

*E. taeniura*IVPP OV 2298, osteological specimen, IVPP.

*
Coelognathus
*

*C. flavolineatus*IVPP OV 2687, osteological specimen, IVPP.

*C. radiatus*IVPP OV 2411–2415, osteological specimens, IVPP.

*
Euprepiophis
*

*Eu. mandarinus*IVPP OV 2675, osteological specimen, IVPP.

*
Oligodon
*

*Ol. ornatus*SYS r001297, 3D model of Impregnated specimens, SYS.

*
Oocatochus
*

*Oo. Rufodorsatus*IVPP OV 2691, osteological specimen, IVPP.

*
Ptyas
*

*P. dhumnades*IVPP OV 2686, osteological specimen, IVPP.

**
Dipsadidae
**

*
Dasypeltis
*

*D. scabra*MHNG 1362.78, lateral and dorsal views of skull (Helfenberger and Schätti 1998). MHNG; AMNH r-31638, 3D model of impregnated specimens, AMNH.

*
Thermophis
*

*T. zhaoermii*BFU R_Th_001. 3D model of impregnated specimen, BFU.
